# Diagnosis and Surgical Treatment of Nasal Squamous Cell Carcinoma in a Vietnamese Pot-Bellied Pig

**DOI:** 10.3390/ani15233432

**Published:** 2025-11-28

**Authors:** Willow R. C. M’Cloud, Edward T. Earley, Brenna R. Pugliese, Garett B. Pearson, Thomas O. C. Ratcliffe, Elena A. Demeter, Ian R. Porter, Rebecca C. McOnie, Eileen S. Hackett

**Affiliations:** 1 College of Veterinary Medicine, Cornell University, 930 Campus Road, Ithaca, NY 14853, USA; wrm79@cornell.edu (W.R.C.M.); ete9@cornell.edu (E.T.E.); tor6@cornell.edu (T.O.C.R.); ed478@cornell.edu (E.A.D.); rebecca.mconie@cornell.edu (R.C.M.); 2 College of Veterinary Medicine, North Carolina State University, 1060 William Moore Drive, Raleigh, NC 27606, USA; brpuglie@ncsu.edu; 3 School of Veterinary Medicine, University of Pennsylvania, 382 West Street Road, Kennett Square, PA 19348, USA; pearsong@vet.upenn.edu

**Keywords:** rhinoscopy, rhinotomy, squamous cell carcinoma, pot-bellied pig, nasal mass

## Abstract

**Simple Summary:**

Squamous cell carcinoma is a common neoplasm of domestic species. Although the rate of metastasis is relatively low, this locally invasive tumor can cause extensive damage and often requires aggressive treatment with surgical excision and adjunctive therapies. The head and perineal/genital regions are most commonly affected, especially in light-skinned animals, indicating a likely role of chronic ultraviolet light injury in cell mutation. Other factors include viral infection and chronic inflammation. There are limited reports of squamous cell carcinoma in pigs and no reports have described successful treatment of nasal squamous cell carcinoma in a pig. The diagnosis of upper respiratory problems in pigs is challenging due to a paucity of research on the subject and examination challenges, with patients typically requiring sedation or anesthesia for evaluation. Advanced imaging modalities are often necessary to identify lesions and direct treatment. In this report, we describe the diagnosis and treatment of a nasal squamous cell carcinoma lesion in a Vietnamese Pot-bellied pig.

**Abstract:**

Background: Though neoplasms in pigs are increasingly recognized, there are no previous clinical descriptions of nasal squamous cell carcinoma. Case Presentation: A 4-year-old 71 kg Vietnamese Pot-bellied pig barrow presented with a history of mild intermittent epistaxis. A mass was identified in the left nasal passage during computed tomography. Rhinoscopic guidance biopsy confirmed squamous cell carcinoma. Surgical excision via dorsal rhinotomy was performed. Nasal swelling prompted repeated computed tomography 1 year later. Mass recurrence was detected, and the pig underwent a second surgical excision with adjunctive cryotherapy. The patient developed a small permanent nasocutaneous fistula, with no evidence of tumor recurrence 1 year following the second surgery. Conclusion: This is the first reported case of a Vietnamese Pot-bellied pig undergoing treatment for nasal squamous cell carcinoma. A multimodal diagnostic and therapeutic approach was applied, including advanced imaging, rhinoscopy, surgical intervention, and vigilant post-operative monitoring, which resulted in successful management and a favorable long-term outcome. Neoplasia should be considered as a differential diagnosis in pigs presenting with nonspecific nasal signs.

## 1. Introduction

Neoplasms in pigs have been recognized with increased frequency in recent years, mainly due to longer lifespans and individualized veterinary care for pet and sanctuary pigs. One comprehensive study of pet Pot-bellied pig specimens submitted for necropsy or biopsy indicated that the most commonly observed neoplasms were leiomyoma, leiomyosarcoma, and hepatocellular carcinoma, with squamous cell carcinoma only identified within nasal or skin samples in 2 of 63 specimens [1]. Squamous cell carcinoma has been reported only sporadically in pigs, in both oral and cutaneous locations of both Pot-bellied and production pigs [2,3,4,5,6,7]. This case report describes the diagnosis and treatment of a Vietnamese Pot-bellied pig with nasal squamous cell carcinoma.

## 2. Case Summary

A 4-year-old 71 kg Vietnamese Pot-bellied barrow presented to the Cornell Hospital for Animals for evaluation of overgrown tusks and mild intermittent epistaxis. On presentation, the patient was bright, alert, and responsive with normal vital parameters. No epistaxis was noted. Examination performed under anesthesia revealed mild asymmetry of the nasal bone and overlying soft tissue at the level of the tusks. The dental examination revealed marked overgrowth of both maxillary and mandibular tusks. Oral endoscopy exposed severe dental calculi and periodontal disease.

**Figure 1:**
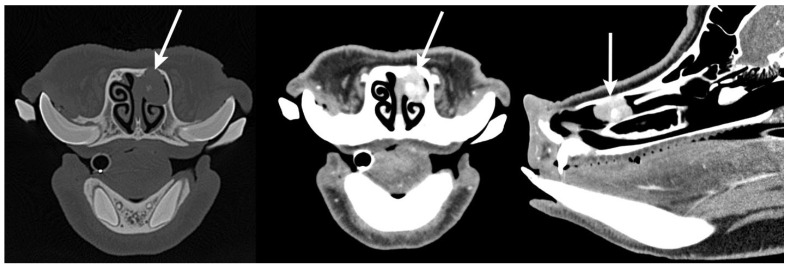
Preoperative transverse and sagittal computed tomography images of the skull from initial presentation highlighting the abnormal soft tissue mass (white arrows) within the left nasal cavity centered on the rostral aspect of the dorsal nasal concha. Images were acquired in both the bone (**Left**) and soft tissue (**Center** and **Right**) windows.

Computed tomography revealed a small, focal, left nasal cavity mass centered on the rostral aspect of the dorsal nasal concha (Figure 1). The mass was approximately 2.4 cm × 1.2 cm × 1.5 cm, with strong, heterogeneous, contrast enhancement and multiple small foci of mineral within the central portion. There was evidence of expansion and thinning of the rostral aspect of the left maxillary bone and left nasal bone, without periosteal new bone formation.

**Figure 2:**
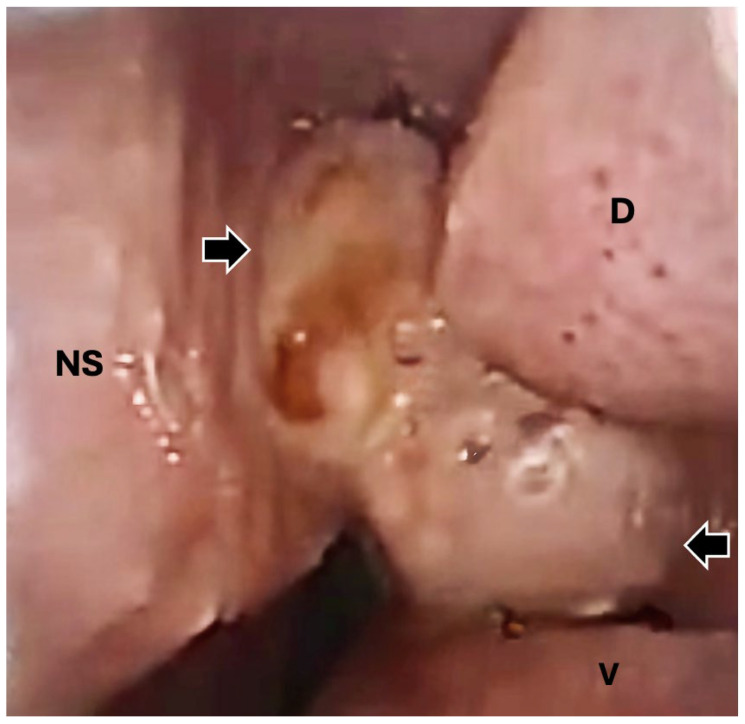
Representative image acquired during rhinoscopy of the left nasal passage revealing the soft tissue mass (indicated by the black arrows). The dorsal (D) and ventral (V) nasal turbinates are indicated, as well as the median nasal septum (NS). Image orientation: dorsal at the top of the image, medial to the left, lateral to the right.

**Figure 3:**
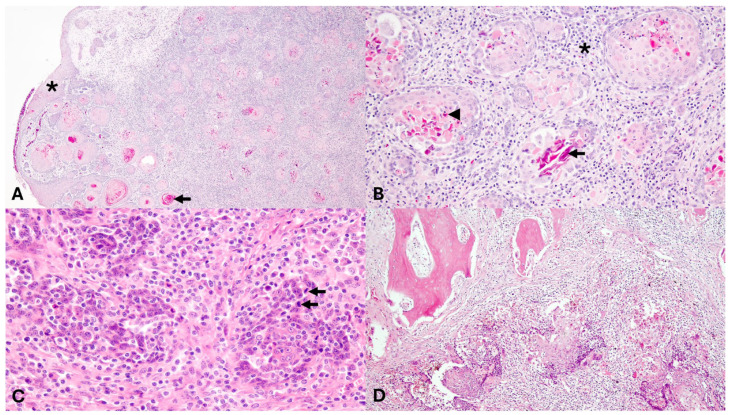
Representative histopathologic images of the left nasal cavity mass biopsy (**A**,**B**) and mass excision (**C**,**D**). (**A**). Nasal stroma expanded by islands and trabeculae of neoplastic cells, often with central keratin (black arrow), enclosed in a dense fibrovascular stroma; * nasal epithelium (H&E, 40×). (**B**). Islands of neoplastic cells containing central keratin (black arrow) and dyskeratotic cells (black arrowhead). The neoplastic cells are polygonal, with distinct borders, abundant eosinophilic cytoplasm, and round nucleus with coarse chromatin. Surrounding the neoplastic islands is a dense stroma (black asterisk) infiltrated by mixed inflammatory cells (H&E; 200×). (**C**). Neoplastic cells organized in poorly distinct clusters with mitotic figures (black arrow) (H&E; 400×). (**D**). Neoplastic islands invading the bone (H&E; 100×).

Rhinoscopy was performed (Arthrex Nanoscope 1.9 mm external diameter, Ambu aScope 4 Broncho 3.8 mm/180°, Arthrex, Naples, FL USA), revealing a soft tissue mass inside the left nasal cavity (Figure 2). A rhinoscopic-guided biopsy of the tissue was collected and sent for histopathology. Following anesthetic recovery, the patient was administered amoxicillin clavulanic acid (13.75 mg/kg PO q12) and carprofen (2.2 mg/kg PO q12) and was discharged to the care of the owner. Histopathology of the nasal mass, processed routinely and stained with hematoxylin and eosin (H&E), showed squamous cell carcinoma. The nasal mass comprised a densely cellular, infiltrative neoplasm composed of neoplastic cells arranged in islands and anastomosing trabeculae on a dense fibrovascular stroma. Neoplastic cells were often arranged in islands centered around concentric whorls of lamellar keratin (keratin pearls) and degenerate cellular debris. Neoplastic cells were polygonal with distinct cell borders, variably distinct intracellular bridges, abundant eosinophilic, often hyalinized cytoplasm, round to irregular nuclei with coarsely clumped chromatin and an indistinct nucleolus. Anisocytosis and anisokaryosis were moderate to severe. There were three mitotic figures in ten 400× fields (2.37 mm^2^). Scattered throughout the stroma were moderate to large numbers of lymphocytes, plasma cells, and neutrophils; lymphovascular invasion was not observed (Figure 3).

The patient was readmitted 3 weeks later for surgical excision of the nasal mass via dorsal rhinotomy. The pig was administered potassium penicillin (22,000 IU/kg IV q6 h), gentamicin (6.6 mg/kg IV q24 h), and pantoprazole (1 mg/kg IV q24 h) perioperatively with a single preoperative dose of ondansetron (0.3 mg/kg IV). Following induction of general anesthesia, the patient was positioned in sternal recumbency. A left infraorbital nerve block was performed with 1.5 mL 0.5% bupivacaine (7.5 mg) with 1:200,000 epinephrine (0.0137 mg) utilizing an intraoral approach by inserting a Tuohy needle into the palpable foramen to a depth of 3.5 cm. The left nostril was irrigated with oxymetzoline nasal spray (2.5 mg). The surgical site (dorsum of the nose) was clipped, aseptically prepared, and draped. An incision was created sharply over the dorsal aspect of the left nasal passage and the nasal bone was exposed. A 1-inch 25 g needle was inserted into the nasal cavity rostral to the nasal mass with rhinoscopic guidance. A 1.5 cm diameter rhinotomy was then created using a Hall air drill equipped with a 5 mm oval burr to allow visualization of the underlying ethmoidal conchae and nasal mass. The mass was then removed. A Foley catheter was inserted into the left nasal passage and the balloon was inflated at the level of the rhinotomy site for hemostasis. Subcutaneous and dermal tissues were opposed with 2-0 Monocryl (Ethicon, Raritan, NJ, USA) in a continuous pattern.

The patient was recovered from anesthesia and administered amoxicillin clavulanic acid and carprofen (same dosage, route and frequency as previously described) after surgery. When appetite decreased 48 h later, the oral medications were discontinued and tulathromycin was administered (2.5 mg/kg SQ) prior to discharge. The excised mass was submitted for histopathology, decalcified, and processed routinely. Findings mirrored the initial biopsy, with the addition of bone invasion by the neoplastic population (Figure 3). Neoplastic cells extended to all tissue margins, consistent with incomplete excision.

The pig was evaluated 6 months following rhinotomy during which time the patient was reportedly doing well at home. On presentation, the patient was bright, alert and responsive. The pig was induced under general anesthesia and rhinoscopy was performed. A small mass of tissue was observed in the left nasal passage at the previous surgical site and biopsy revealed mild, multifocal, chronic lymphoplasmacytic, neutrophilic, and eosinophilic rhinitis without evidence of neoplasia. The patient was managed on perioperative carprofen and amoxicillin clavulanic acid (same dosage, route and frequency as previously described) and was discharged the following day. 

**Figure 4:**
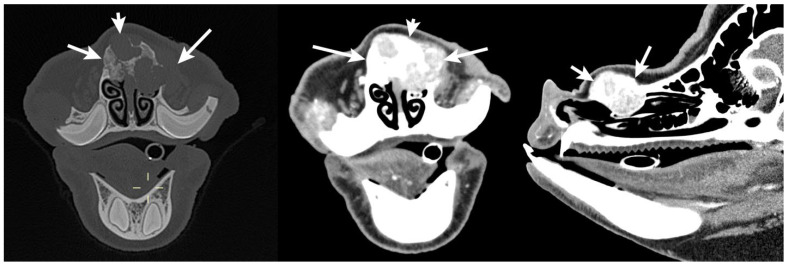
Transverse and sagittal computed tomography images of the skull 1 year post rhinotomy highlighting the recurrence of the mass of the left nasal passage (white arrows) with involvement of the nasal septum and locally extensive severe lysis and proliferation of both the left and right nasal bone. Images were acquired in both the bone (**Left**) and soft tissue (**Center** and **Right**) windows.

The patient returned for recheck evaluation 1 year post rhinotomy. The owners reported a slowly enlarging lump on the snout at the previous surgical site. The pig underwent general anesthesia for computed tomography which revealed a soft tissue mass in a similar location to the original nasal tumor. The mass extended to the nasal septum with locally extensive severe lysis and proliferation of the left and right nasal bones (Figure 4). Recurrence of squamous cell carcinoma was suspected. Two days later, the patient underwent general anesthesia and excision of the nasal mass. The patient was positioned in dorsal recumbency for jugular catheterization (5Fr 13 cm double lumen catheter, Mila International, Hebron, KY, Australia) with ultrasound guidance and then moved to sternal recumbency for bilateral infraorbital nerve blocks performed with 3 ml 0.5% bupivacaine (15 mg) with 1:200,000 epinephrine (0.0274 mg). The dorsum of the nose was then clipped, scrubbed, and draped using standard aseptic technique. A midline incision was made over the nasal passages dorsally. The underlying subcutaneous tissues were bluntly dissected and the periosteum was elevated to expose the nasal bone. A rhinotomy was created in the region overlying the nasal mass with a Hall Air drill equipped with a 5 mm oval burr (Conmed Corporation, Utica, NY, USA). This incision exposed the underlying ethmoidal conchae and nasal mass, and the mass and a portion of the nasal septum were removed. Following removal, cryotherapy of the region was performed using liquid nitrogen applied in 2 freeze–thaw cycles. Subcutaneous and dermal tissues were opposed with 2-0 Monocryl (Ethicon) in a continuous pattern. Red rubber catheters were positioned in both nasal passages to support nasal breathing during anesthetic recovery.

The patient was initially managed on a lidocaine CRI (25μg/kg/min IV for 24 h), along with flunixin meglumine (1.1 mg/kg IV q24 h) and pantoprazole (1 mg/kg IV q24 h) for 4 days. The pig continued to recover in hospital and the IV catheter was then removed and the patient was discharged 5 days post-operatively.

Histopathology of the mass confirmed recurrence of squamous cell carcinoma. The neoplasm was described as infiltrative, highly cellular, unencapsulated, and composed of islands and trabeculae of neoplastic epithelial cells with disorderly maturation, supported by a collagenous stroma infiltrated by lymphocytes and plasma cells. Neoplastic keratinocyte cells were polygonal with distinct borders and abundant eosinophilic fibrillar cytoplasm. Nuclei were round to oval with open chromatin and up to two prominent nucleoli. Anisokaryosis and anisocytosis were moderate. Mitoses were six in ten 400× fields (2.37 mm^2^) and existed outside the basal layer. Neoplastic cells were keratinizing with multiple areas of islands centered around concentric whorls of lamellar keratin (keratin pearls) and degenerate cellular debris. Frequently, individual keratinocytes had hypereosinophilic cytoplasm and condensed nuclei (dyskeratosis). Multifocally, large nodules of neoplastic cells invaded the bone. Lymphovascular invasion was not noted, and neoplastic cells extended to the tissue margins, consistent with incomplete excision.

A total of 10 days after discharge (15 days post-surgery), the patient presented for suspected incisional dehiscence. Under stall-side sedation, the area was lightly debrided. Sedation and light debridement were repeated once more 4 days later, and the patient was discharged. The pig continued healing with continued daily wound care and a portion of the nasal incision remained open.

**Figure 5:**
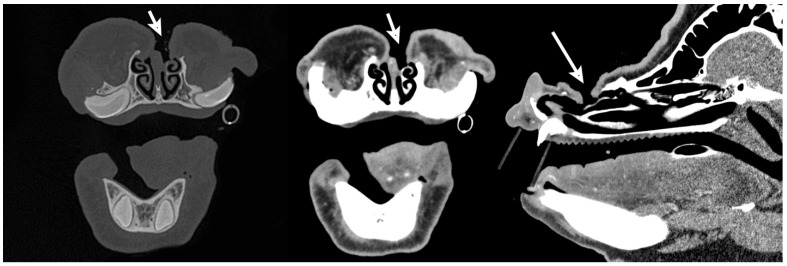
Transverse and sagittal computed tomography images of the skull 1 year after the second rhinotomy with the partial nasal septum resection. Computed tomography highlights the previous site of the mass with no signs of mass recurrence and the persistent nasocutaneous fistula into the left nasal cavity (white arrows). Images were acquired in both the bone (**Left**) and soft tissue (**Center** and **Right**) windows.

**Figure 6:**
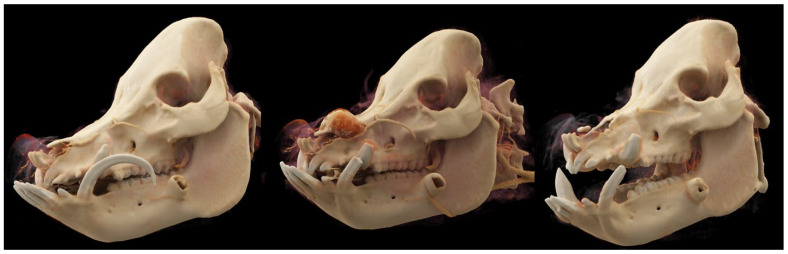
Three-dimensional reconstruction of the patient’s skull on initial presentation (**Left**), at recheck at 1 year with recurrence of the mass and involvement of the nasal bones (**Center**), and at final recheck at 1 year post second rhinotomy and partial nasal septum resection (**Right**).

The pig was re-evaluated 1 year later. On presentation, the patient was bright, alert, and responsive, with vital parameters within normal limits. An approximately 5 mm nasocutaneous fistula was visible communicating with the left nasal passage and no evidence of inflammation or infection was present. No recurrence of nasal neoplasia was apparent on computed tomography examination. The bony margins surrounding the previous rhinotomy site were smooth (Figure 5). The pig recovered well from anesthesia and was managed on omeprazole (1 mg/kg PO q24 h), amoxicillin clavulanic acid, and carprofen (same as previous dosing) perioperatively. The patient was comfortable and was discharged the following day. Figure 6 summarizes the computed tomography examinations performed at three timepoints during treatment with 3D renderings.

## 3. Discussion

This report describes a Vietnamese Pot-bellied pig with intermittent sneezing and epistaxis secondary to nasal squamous cell carcinoma. An interdisciplinary diagnostic approach utilized oral examination, computed tomography, and rhinoscopic biopsy to definitively identify the nasal mass prior to surgical excision. Rhinoscopy was particularly beneficial for targeted biopsy which informed treatment decisions. This comprehensive diagnostic approach allowed rapid identification and diagnosis and facilitated surgical planning. 

Squamous cell carcinoma is a malignant neoplasm of the epidermis [8]. It is a common neoplasm of many domestic species, including cats, dogs, cows, and horses, and has been described in goats and pigs [2,3,6,7,8,9,10,11,12,13]. In small animals, these tumors most commonly occur in older cats and dogs over the age of 10, with predisposition to development of squamous cell carcinoma lesions in areas of the head, especially in thin-haired light-coated animals [8]. Similarly, in horses and cows, the most common presentation involves lesions in non-pigmented skin around the eyes, perineal, or genital regions, or malignant transformation of wound sites due to chronic inflammation or physical irritation [12,13]. A few reports of squamous cell carcinoma in pigs exist, describing aggressive oral lesions and diffuse cutaneous lesions in production breed pigs [2,3,6,7]. Accepted etiologies behind development of squamous cell carcinoma include mutations resulting from chronic ultraviolet light exposure, chronic inflammation or physical irritation, and viral infection [8,12]. It is unlikely that ultraviolet light exposure played a significant role in the present case given the location; therefore chronic inflammation and irritation or viral infection might have contributed.

Limited information is available on diagnosis or treatment of nasal squamous cell carcinoma in Pot-bellied pigs [1]. There are two reports of aggressive oral squamous cell carcinoma lesions in Pot-bellied pigs [2,3]. Kleinschmidt et al. reported on a 10-year-old pig presented for several months of weight loss and difficulty eating, in which an ulcerated, infiltrative mass was identified affecting the left oral cavity and palate resulting in euthanasia [3]. Another report describes an 18-year-old Pot-bellied pig that was treated initially for severe periodontal disease, abscesses and oronasal fistula and, 18 months later, was readmitted with a large mass affecting the right mandible and intermandibular space [2]. The pig euthanized after a 3-month treatment with antibiotics and anti-inflammatories [2]. In the limited reports of pigs undergoing treatment for squamous cell carcinoma lesions, two describe treatment of cutaneous squamous cell carcinoma with electrochemotherapy [6,7]. Weissman et al. described successful treatment of diffuse cutaneous squamous cell carcinoma lesions in a 9-year-old pot-bellied pig with eight sessions of electrochemotherapy using intravenous bleomycin [7]. This pig reportedly tolerated treatment well and, at 18 months post-diagnosis, was in sustained partial remission, with a new lesion growing in an untreated area. Gould et al. reported treatment of periocular and pinnal cutaneous squamous cell carcinoma in an American Yorkshire pig using three sessions of electrochemotherapy with bleomycin, followed by longer-term management of tumors with CO_2_ laser ablation and cryotherapy [6]. To our knowledge, the present case is the first report of diagnosis and treatment of nasal squamous cell carcinoma in a Pot-bellied pig.

Nasal squamous cell carcinoma has been reported in dogs, most commonly affecting the nasal planum and occasionally the rostral nasal septum [14]. In dogs with squamous cell carcinoma of the rostral nasal septum, rostrolateral rhinotomy is reported to result in more complete resection and good cosmetic outcome [14]. Other reported therapies include surgical resection combined with radiation therapy, CO_2_ laser ablation, cryosurgical ablation, and radiation therapy [15,16,17,18]. In cats, squamous cell carcinoma of the nasal planum makes up around half of reported nasal tumors and reported treatment protocols include surgical excision, radiation therapy photodynamic therapy, and electrochemotherapy [19,20,21,22,23,24,25,26]. In the present report, treatment with surgical resection alone resulted in recurrence within 1 year of surgery; therefore, cryosurgical ablation was combined with surgical resection in subsequent treatment to achieve a 1-year disease-free interval confirmed by computed tomography. 

Identification of nasal masses within the nasal passages is facilitated by advanced imaging. In the present case, both computed tomography and rhinoscopy combined with biopsy were utilized to obtain a diagnosis and create a treatment plan. A study by Finck et al. showed that computed tomography appeared to be more reliable than rhinoscopy in diagnosing nasal masses as a first-line diagnostic tool in dogs and cats affected by nasal tumors [27]. In the present case, periodic rechecks with computed tomography and rhinoscopy were beneficial in guiding continued care and recommendations. Although the nasal biopsy indicated presence of inflammatory tissue at his initial 6-month recheck, repeat computed tomography 1 year later was recommended due to external nasal swelling. At that time, a mass was identified in a similar location indicating recurrence and prompting surgical removal. The pig was closely monitored and recheck computed tomography performed 1 year after the second rhinotomy showed no further evidence of recurrence. Serial computed tomography, complimented by rhinoscopy if appropriate, is recommended in pigs with nasal tumors to assess the region and signal if treatment is indicated.

## 4. Conclusions

This case report describes successful diagnosis and management of a locally invasive squamous cell carcinoma of the nasal cavity in a Vietnamese Pot-bellied pig. Pursuing advanced diagnostics including rhinoscopy and computed tomography allowed prompt diagnosis of the nasal mass and subsequent initiation of treatment. This case also highlights the importance of serial recheck examinations and imaging to monitor for recurrence and treatment if necessary. The outcome in this case was positive, requiring repeated surgical excision, and the pig continues to have a good quality of life without additional recurrence.

## Data Availability

The original contributions presented in the study are included in the article, further inquiries can be directed to the corresponding author.
